# The genetic risk of Alzheimer’s disease beyond *APOE ε4:* systematic review of Alzheimer’s genetic risk scores

**DOI:** 10.1038/s41398-018-0221-8

**Published:** 2018-08-24

**Authors:** Hannah Stocker, Tobias Möllers, Laura Perna, Hermann Brenner

**Affiliations:** 1 Network Aging Research, University of Heidelberg, Bergheimer Straße 20, Heidelberg, 69115 Germany; 2 Division of Clinical Epidemiology and Aging Research, German Cancer Research Center, Im Neuenheimer Feld 581, Heidelberg, 69120 Germany; 3 Medical Faculty, University of Heidelberg, Heidelberg, 69120 Germany

## Abstract

The ε4 allele of Apolipoprotein E (*APOE*) is the strongest known genetic risk factor of Alzheimer’s disease (AD) but does not account for the entirety of genetic risk. Genetic risk scores (GRSs) incorporating additional genetic variants have been developed to determine the genetic risk for AD, yet there is no systematic review assessing the contribution of GRSs for AD beyond the effect of *APOE* ε4. The purpose of this systematic PRISMA (Preferred Reporting Items for Systematic Reviews and Meta-analyses)-based review was to summarize original research studies that have developed and validated a GRS for AD utilizing associated single nucleotide polymorphisms (SNPs). The PubMed and Web of Science databases were searched on April 6, 2018 and screening was completed on 2018 citations by two independent reviewers. Eighteen studies published between 2010 and 2018 were included in the review. All GRSs expressed significant associations or discrimination capability of AD when compared to clinically normal controls; however, GRS prediction of MCI to AD conversion was mixed. *APOE* ε4 status was more predictive of AD than the GRSs, although the GRSs did add to AD prediction accuracy beyond *APOE* ε4. GRSs might contribute to identifying genetic risk of AD beyond *APOE*. However, additional studies are warranted to assess the performance of GRSs in independent longitudinal cohorts.

## Introduction

Alzheimer’s disease (AD) is the most common form of dementia and is a critical public health issue across the globe^1^. The etiology of the disease is thought to be a complex interaction between genes, environmental and lifestyle factors^2^. The heritability of late-onset AD has been estimated around 74%^3^. The strongest known genetic risk factor for AD is the ε4 allele of Apolipoprotein E (*APOE* ε4), but large-scale genome-wide association studies (GWASs) have identified additional genetic loci associated with AD^4–7^.

The largest GWAS meta-analysis concerning AD to date (*N* = 74,046), The International Genomics of Alzheimer’s Project (IGAP), has confirmed at least 20 genetic loci in addition to *APOE* genotype to be associated with AD^8^. The IGAP is a large two-stage study based upon GWASs on individuals of European ancestry. In stage 1, IGAP used genotyped and imputed data on 7,055,881 single nucleotide polymorphisms (SNPs) to meta-analyze four previously-published GWAS datasets consisting of 17,008 AD cases and 37,154 controls. In stage 2, 11,632 SNPs were genotyped and tested for association in an independent set of 8572 AD cases and 11,312 controls. Finally, a meta-analysis was performed combining results from stages 1 and 2^8^.

IGAP consortia samples have greatly contributed to advancing genetic risk scores (GRSs) for AD, a strategy developed to deal with the relatively small magnitudes of association of the additional genetic loci for AD. GRSs determine the genetic risk for a disease through the composite consideration of many individual effects of genetic loci, which when considered collectively could account for substantial differences in risk of disease^9^. Thus, GRSs might present an effective strategy to combine the relatively smaller effects of AD associated loci to assess genetic risk beyond *APOE* ε4 status. However, the predictive value and methodologies of GRSs vary greatly between studies. For example, Escott-Price et al. analyzed more than 200,000 SNPs, including *APOE* resulting in an area under the curve (AUC) value of 0.84^10^, while Tosto et al. used only 21 SNPs excluding *APOE* and reported an AUC of 0.57^4^.

Assessing the genetic contribution of GRSs to AD is of importance to better identify those with a higher susceptibility to AD and, eventually, enable targeted prevention strategies. To date there is no systematic review assessing GRSs for AD available. The aim of this literature review was to summarize original research studies that have developed and validated a GRS for AD utilizing associated SNPs.

## Methods

The literature review was planned and performed using methods specified in the Preferred Reporting Items for Systematic Reviews and Meta-Analyses (PRISMA) statement for reporting systematic reviews and meta-analyses^11^. Searches were completed in the PubMed and Web of Science databases (see Supplementary Table 1 for search strategies) on April 6, 2018 following the inclusion criteria: (1) presence of the evaluation of a defined GRS for AD incorporating genetic variants (specifically SNPs); (2) SNPs in GRS directly associated to AD; (3) AD diagnosis as the main outcome; (4) adult population of European descent; and (5) English or German language manuscripts. Specifically excluded were studies with all-cause dementia as an outcome where AD could not be specified as the outcome of interest. Searches were not limited to a specific time period. Based on the eligibility criteria, two reviewers (HS, TM) independently performed the study selection and in case of discrepancy discussion and further review of the issue followed in consultation with a third reviewer (LP).

### Data extraction

The reviewers (HS and TM) extracted the following data from the included articles: (1) type of study; (2) validation data set information (study name, sample size, case number, mean age & sex distribution); (3) training data set information (study name, sample size & case number); (4) number of SNPs in the GRS; (5) type of GRS used (weighted or unweighted); (6) association between GRS and AD diagnosis; (7) the covariates considered; and (8) whether *APOE* was included in GRS. Additionally, information regarding the specific SNPs used in each of the GRSs was extracted including the name, location, gene, and association (odds ratio (OR) or log hazard ratio (HR)).

### Quality assessment

The quality of the included studies was assessed independently by two reviewers (HS & TM) through an adapted version of the Newcastle-Ottawa scale (NOS), which assesses the quality of non-randomized studies based on three main categories: (1) the selection of the study groups; (2) the comparability of the groups; and (3) the ascertainment of either the exposure or outcome of interest. This tool was chosen because of the type of studies included^12^. The assessment tool was adapted to best fit the included studies based upon our inclusion criteria, where the exposure was genetic risk, the outcome of interest was AD diagnosis and the important covariates were age, sex and *APOE* e4 status. A coding manual was developed to ensure consistency and understanding of assessment. A point was awarded in each of nine categories if the study met the outlined criteria^13^.

## Results

**Fig. 1:**
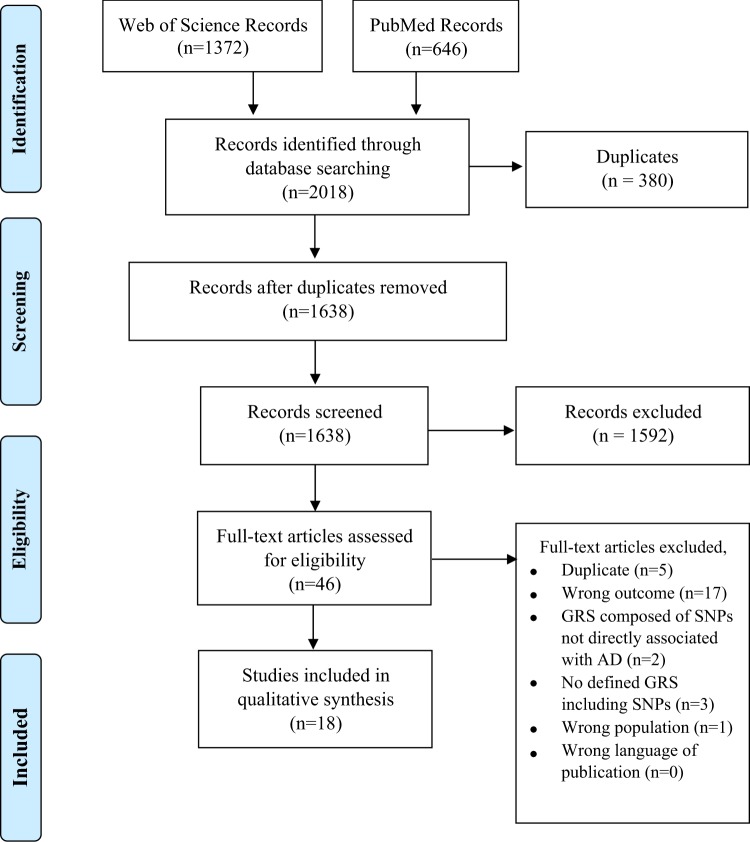
Flowchart of the process of literature search and extraction of studies meeting the inclusion criteria

The initial database searches identified 1372 articles from Web of Science and 646 articles from PubMed resulting in a total of 2018 articles. Of the 1638 articles that remained after duplicates were removed (*n* = 380), 1592 were excluded because of irrelevance to the topic (Fig. 1). Strict inclusion criteria, as outlined above, were applied to the full text of 46 articles. Of these, 18 met the full set of criteria (Table 1)^4,14–29^. All articles were published between 2010 and 2018. PRISMA guidelines were followed throughout the review and reporting process, please see Supplementary Table 2 for a completed PRISMA checklist.

### Study characteristics

An overview of the study characteristics can be seen in Table 1. The majority of the studies used a case-control study design comparing AD cases to clinically normal controls^4,10,14,17,20–23,27,28^, the remaining studies utilized a longitudinal cohort^19,24,25,29–31^, nested case-control^15^ or cross-sectional design^26^ (Table 1). All training samples included individuals of European descent and ranged from 192^20^ to 74,046 (IGAP meta-analysis)^8^ individuals. The validation samples were also of European descent and ranged from 204^26^ to 19,687^19^ individuals. The majority of studies used IGAP consortia samples for the training and validation sets (Table 1). The selection of SNPs and corresponding magnitudes of associations were derived from a training set while the resulting GRS was assessed in a validation set. Six studies used data sets not associated with IGAP for GRS validation^20,23–25,27,31^. Three studies used a validation sample or group of participants of European descent with a family member afflicted with AD and therefore were not completely representative of a general population of European descent^4,14,28^. While the majority of included studies compared clinically normal controls to AD participants, two studies examined the ability of a GRS to predict the transition from mild cognitive impairment (MCI) to AD^24,25^, and one study examined both^31^.

**Table 1:** Study characteristics

First Author, Year	Type of study	Training set			Validation set				
		Study name	Total N	Cases	Study name	Total N	Cases	Age (mean)	%Male
*GRS for AD diagnosis: clinically normal to AD comparison or transition*									
Ahmad, 2018^29^	cohort	IGAPˠ,	74,046	25,580	Rotterdam Studyˠ	8,893	1,270	84*	41%
Van der Lee, 2018^30^	cohort	IGAPˠ & various GWAS	—	—	Rotterdam Studyˠ	12,255	1,262	68	42%
Cruchaga, 2018^28^	case-control	IGAPˠ,	74,046	25,580	Knight-ADRC, ADNIˠ, NIA-LOADˠ	3,836	2,825	72	43%
Chaudhury, 2018^27^	case-control	IGAP stage 1ˠ	54,162	17,008	UK Centers	844	408	57	52%
Tan, 2018^31^	cohort	IGAP stage 1ˠ	54,162	17,008	NACC	1,652	428	73	41%
Tosto, 2017^4^	case-control	IGAPˠ,	74,046	25,580	NIA-LOADˠ	4,792	2,128	74	38%
Escott-Price, 2017^10^	case-control	IGAP stage 1ˠ	54,162	17,008	GERADˠ	1,594	1,011	—	—
Desikan, 2017^15^	nested case-control	IGAP stage 1ˠ	54,162	17,008	ADGCˠ	15,795	6,409	76	40%
Tosto, 2016^14^	case-control	IGAPˠ	74,046	25,580	NIA-LOADˠ	2,567	1,243	77	39%
Chouraki, 2016^19^	cohort	IGAPˠ	74,046	25,580	IGAPˠ	19,687	2,782	76	39%
Lupton, 2016^23^	case-control	IGAP stage 1ˠ	54,162	17,008	AddNeuroMed	202	99	64	44%
Yokoyama, 2015^20^	case-control	UCSF MAC	192	59	UCSF MAC	276	126	80	55%
Sleegers, 2015^21^	case-control	IGAPˠ & various GWAS	—	—	Flanders-Belgianˠ	2,181	1,162	72	41%
Escott-Price, 2015^17^	case-control	IGAP stage 1ˠ	43,708	14,831	IGAP subsetˠ	4,603	3,049	—	—
Sabuncu, 2012^26^	cross sectional	ADNIˠ	197	—	ADNIˠ	204	100	76	52%
Biffi, 2010^22^	case-control	Various GWAS	—	—	ADNIˠ	383	168	75	41%
*GRS for AD diagnosis Mild cognitive impairment to AD transition*									
Lacour, 2017^24^	cohort	IGAP & various GWASˠ	—	—	AgeCoDe, DCN, ACE, ADC	3,216	790	73	46%
Rodriguez-Rodriguez, 2013^25^	cohort	Various GWASˠ	—	—	Spanish clinical cohort	288	118	74	51%

ˠ *IGAP* associated data set, *ACE* Fundacio ACE Barcelona, *mean age of Alzheimer's disease onset in cases, *ADC* Amsterdam Dementia Cohort, *ADNI* Alzheimer’s Disease Neuroimaging Initiative, *AgeCoDe* German study on Aging, Cognition and Dementia in primary care patients, *DCN* German Dementia Competence Network, *GERAD* Genetic and Environmental Research in Alzheimer’s Disease, *GWAS* Genome-wide association study, *IGAP* International Genomics of Alzheimer’s Project, *Knight-ADRC* Knight Alzheimer’s disease research center, *NACC* National Alzheimer’s Coordinating Center, *NIA-LOAD* National Institute on Aging Genetics Initiative for Late-Onset Alzheimer’s Disease Family Study, *UCSF MAC* University of California, San Francisco Memory and Aging Center

AD participants within the training sets of the included studies met National Institute of Neurological and Communicative Disorders and Stroke and Alzheimer’s Disease and Related Disorders Association (NINCDS/ADRDA) criteria for probable AD, were autopsy confirmed, or met consensus criteria for AD^32^. Similarly in all validation sets, AD participants met NINCDS/ADRDA criteria or were confirmed through autopsy with the exception of two studies^20,24^.

### GRS Construction

All included studies developed and validated a defined GRS for AD comprised of varying AD associated SNPs. The number of SNPs included in the GRSs ranged from 5^22^ to 359,500^17^ (Table 2). SNP inclusion in the GRSs was based on two approaches: (1) selection from genome-wide significant results of previous GWAS (mainly IGAP meta-analysis)^4,14,19–22,24,25,27–31^ or (2) following *p*-value cutoffs including many SNPs^10,15,17,23,26^ (Table 2). The specific SNPs used in the included studies can be found in Supplementary Table 3 with information regarding location and associated gene. All studies utilized a weighted GRS as outlined by Purcell et al.^9^. Finally, *APOE* was either considered as a covariate^4,14,19,22,24,25,28,31^, included in the GRS^10,15,17,20,21,26,27^, not included and not considered as a covariate^23,29,30^.

**Table 2:** GRS results with comparison to APOE

First Author, year	#SNPs	AUC^APOE^ (95%CI) or ± SE	AUC^GRS^ (95%CI) or ± SE	OR^APOE^ (95%CI) or ± SE	OR^GRS^ per SD (95%CI)	HR^APOE^ (95%CI)	HR^GRS^ per SD (95%CI)	Covariates considered
*GRS for AD diagnosis: clinically normal to AD comparison or transition*								
Chaudhury, 2017^27^	28	0.65	0.73^*apoe*^ sEOAD	—	—	—	—	—
Escott-Price, 2017^10^	205,068 (*p* < 0.5)	—	0.84^*apoe*^ (0.81–0.86)	—	—	—	—	10 principal components
Escott-Price, 2015^17^	359,500 (*p* < 0.5)	0.69 (0.67–0.70) (ε2 + ε4 weighted count)	0.75^*apoe*^ (0.73–0.79) 0.72^*apoe*+IGAPSNPs^ (0.70–0.73)	—	—	—	—	Age, sex, country of origin, 3 principal components
Yokoyama, 2015^20^	17	0.63 ± 0.03	0.62 ^apoe^ ± 0.04	—	—	—	—	Age, sex
Cruchaga, 2018^28^	18	—	sLOAD: 0.67 (0.65–0.69)		sLOAD: 1.41* (*p* = 1.3e−3) fLOAD: 2.01* (*p* = 1.3e−6) sEOAD: 2.2* (*p* = 1.6e−6) all 3^rd^ to 1^st^ tertile	—	—	Age, sex, *APOE*
Lupton, 2016^23^	4,431 (*p* < 0.001)	0.8	0.75	2.41* ± .49, (*p* = 1.6e−5)	1.51* ± .24, *p* = .01	—	—	Age, sex, 4 ancestry components
Sleegers, 2015^21^	22	0.67 (age weighted)	0.60 0.70^*apoe*^	—	2.32*^apoe^ (2.08–2.58) per unit	—	—	Age, sex
Tosto, 2017^4^	21	NA	0.57 (0.55–0.59)	4.87* (4.22–5.63)	1.31* (1.23–1.40)	—	—	Age, sex, familial relationships, study center, *APOE*
Tosto, 2016^14^	22	—	—	4.47* (3.87–5.17)	2.85*^NA^ (2.05–3.97)	—	—	Age, sex, familial relationships, study center, education, *APOE*
Sabuncu, 2012^26^	26 (*p* < 10^–5^)	—	—	—	2.06*^apoe^,(*p* < 0.001) 1.44*, (*p* < 0.01)	—	—	Age, sex, education
Biffi, 2010^22^	5	—	—	2.07* (1.67–2.56)	1.14* (1.04–1.25) per quartile	—	—	Age, sex, hypertension, education, alcohol abuse, smoking, *APOE*
Ahmad, 2018^29^	20	—	—	—	—	—	1.27* (1.19–1.34)	Baseline age, sex
Van der Lee, 2018^30^	23	—	—	—	—	—	1.11 (0.97–1.28) 3^rd^ to 1^st^ tertile	Age at inclusion, squared age at inclusion, sex, main genetic effects
Tan, 2018^31^	31	—	—	—	—	—	2.36* (1.38–4.03) 84 to 16 percentile	Baseline age, sex, education, *APOE*
Desikan, 2017^15^	31 (*p* < 10^−5^)	—	—	—	—	—	3.34*^apoe^ (2.62–4.24) 10^th^ to1^st^ decile	Age, sex, genetic components
Chouraki, 2016^19^	18	—	—	—	—	2.08* (1.92–2.26)	1.17* (1.13–1.21)	Age, sex, familial relationships, study center, education, *APOE*
*Mild cognitive impairment to AD conversion*								
Tan, 2018^31^	31	—	—	—	—	—	1.17* (1.02–1.35) 84 to 16 percentile	Baseline age, sex, education, *APOE*
Lacour, 2017^24^	18	—	—	—	—	2.20* (1.88–2.53)	1.18 (0.37–2.0)	Age, sex, education, *APOE*
Rodriguez-Rodriguez, 2013^25^	8	—	—	4.56* (2.23–9.38)	1.32 (0.57–3.06) 3^rd^ to 1^st^ tertile	—	—	Age, sex, *APOE*

All GRS shown are weighted. *APOE* scores were defined as binary variables, presence of 1 or 2 ε4 alleles vs. none, unless otherwise noted. GRS scores did not include *APOE* unless noted (^*apoe*^)

^*^indicates statistically significant result

*NA* indicates that units for OR were not available, *AD* Alzheimer’s disease, *APOE* apolipoprotein E, *AUC* area under the receiver operator curve, *fLOAD* familial late onset Alzheimer’s disease, *GRS* genetic risk score, *HR* hazard ratio, *MDS* multidimensional scaling, *OR* odds ratio, *SD* standard deviation, *SE* standard error, *sEOAD* sporadic early onset Alzheimer’s disease, *sLOAD* sporadic late onset Alzheimer’s disease

### GRS and AD association

#### Clinically normal to AD comparison or transition

The GRSs were found to be predictive of AD status or of AD conversion in all included studies, although varied magnitudes of association or discrimination abilities were found. Eight studies measured the disease prediction accuracy of the GRS using the area under the receiver operating characteristic curve^4,16,17,20,21,23,27,28^. Of which, five GRSs included *APOE* with an AUC range: 0.62–0.84^10,17,20,21,27^ and four GRSs excluded *APOE* with an AUC range: 0.57–0.75^4,21,23,28^. Five studies used time-to-event analysis to evaluate the risk for developing AD^15,19,29–31^. Of those studies, one study included *APOE* in the GRS and reported a 3.34 fold increased risk of AD in individuals in the 10^th^ decile of the GRS compared to the 1^st^ decile ^15^ and the remaining four studies did not include APOE in the GRS with HR range: 1.11 (per SD) – 2.36 (84–16 percentile)^19,29–31^. Seven studies expressed statistically significant associations between the GRS and AD with odds ratios, mainly (*n* = 4) per standard deviation (SD) increase in GRS. Only two GRSs included *APOE* (OR range: 2.06– 2.32)^21,26^, while the remaining five GRSs excluded *APOE* (OR range: 1.14–2.85)^4,14,22,23,28^. Four studies which reported ORs also reported AUC values and were included in the description above^4,21,23,28^ (Table 2). For more detailed information including specific covariates considered in each GRS, please see Table 2.

The ability of the GRS in addition to *APOE* ε4 status to determine AD was investigated in many of the included studies. Possessing one or more *APOE* ε4 allele expressed greater discrimination ability than the GRSs (which excluded *APOE*); however, including *APOE* in the GRS increased AD prediction accuracy (Table 2).

### Mild cognitive impairment to AD conversion

One study expressed a statistically significant result in the prediction of AD conversion from MCI, when comparing the 84^th^ to 16^th^ percentile (HR: 1.17, 95%CI: 1.02–1.35)^31^. The remaining two studies that examined the ability of the GRS to predict MCI conversion to AD did not express a statistically significant result^24,25^. Rodriguez-Rodriguez et al. reported that the GRS was not significantly associated with risk of AD conversion when comparing the 3^rd^ to the 1^st^ tertile of the GRS (OR: 1.32, 95%CI: 0.57–3.06). The hazard model from Lacour et al. also lacked a significant result (HR: 1.18, 95%CI: 0.37–2.0). Nevertheless, *APOE* ε4 status was predictive of MCI to AD conversion (Table 2).

### Quality assessment

The results of the quality assessment using the adapted NOS are shown in Supplementary Table 4. Included studies were of high quality with a mean score of 7.2 stars (maximum 9) and a range of 5–8 stars. All case-control studies included adequate case and control definitions^4,10,17,20–23,27,28^, the vast majority included used representative samples^10,15,17,19,21–26,28–31^, and controlled for age, sex as well as accounted for *APOE ε4*^4,14,15,17,19,21–26,28–31^. All included studies attained adequate and appropriate measure of the exposure (genetic risk) and outcome (AD diagnosis).

## Discussion

This systematic review outlined and compared the existing GRSs for AD and found that the available GRSs resulted in statistically significant associations or disease prediction accuracy of AD when compared to clinically normal adults. However, results were mixed in predicting MCI to AD conversion and the GRSs were less predictive of AD than *APOE* ε4 status. Nevertheless they still contributed to disease prediction accuracy beyond *APOE* ε4.

### Evolution of the GRS (clinically normal to AD)

Since 2010 GRSs for AD have advanced to include a higher number of SNPs, longitudinal assessment, pathological diagnosis, and have witnessed an increased rate of development after the publication of the IGAP meta-analysis in 2013. In three of the more recent studies, liberal GRSs (including thousands of SNPs associated to AD) were applied in addition to a conservative GRS (including only the few genome-wide significant SNPs)^10,17,23^. Conservative GRSs have been the main approach since the development of GRSs for AD, but this may begin to shift. This is evident when comparing the first GRS for AD, which included five SNPs^22^ to one of the most recent GRSs, which included 205,068 SNPs^10^. The liberal GRSs have illustrated greater disease prediction accuracy (AUC range: 0.75–0.84)^10,17^ than the conservative GRSs (AUC range: 0.57–0.72)^4,17,20,21,28^, suggesting that the conservative approach may be too cautious and that a more liberal method may increase disease prediction accuracy. However, an extremely liberal approach, including all SNPs with *p*-value < 0.5^10,17^, may also have led to inclusion of many noninformative SNPs, and even better prediction accuracy might be achieved with an intermediate approach (not too restrictive but also not too liberal criteria). This has been demonstrated by two studies that have reported an increase in the ability of a GRS (also based on IGAP data) to differentiate between clinically normal controls and AD cases when including all SNPs *p*-value < 0.01 or < 0.001 compared to more conservative inclusion, but that after these critical points, discrimination ability plateaued and decreased^23,33^. It is important to note however that these studies used small validation sets, therefore warranting additional confirmation in larger sample sizes in future studies.

Also, GRSs have evolved to validation within a longitudinal study-design in addition to the previous case-control design. In order to confirm the ability of the GRS to predict AD diagnosis, the use of a longitudinal cohort is superior to a case-control study design due to the progressive nature and age dependence of the disease^15^. Five of the most recent studies examined the ability of the GRS to predict AD from clinically normal individuals at baseline or as a comparison and were published from 2016–2018^15,19,29–31^. All studies reported significant results except one^30^. The main limitation of these studies is that both training and validation sets were a part of IGAP in all except one^31^. Additional longitudinal studies investigating the prediction capabilities of a GRS for AD in independent data sets are necessary to assess the plausibility of the GRS in genetic risk assessment.

Only two GRSs to date have been validated in a data set of exclusively pathologically confirmed AD cases^10,27^. Previous studies mainly utilized NINCDS/ADRDA criteria, which have been shown to have a sensitivity of 81% and specificity of 70% in determining AD^34^. Although the NINCDS/ADRDA criteria are widely used in research, autopsy confirmation of AD is the gold standard. Escott-Price et al. showed more accuracy in disease prediction in pathologically confirmed cases than in other validation sets without explicit autopsy confirmation, which points to possible AD misdiagnoses in NINCDS/ADRDA confirmed cases^10^. However this finding needs further replication.

Finally, before the IGAP meta-analysis was published only three studies had been published investigating the use of GRS for AD. Since publication, 15 GRS studies have been published, 11 of which have utilized the IGAP data for the training and validation sets (Supplementary Table 5). Overlap was present in 11 studies, of which only six studies discussed the overlap with five completing additional analysis excluding the overlapping individuals or statistically accounting for overfitting (Supplementary Table 5). The use of overlapping training and validation sets presents a source of possible overfitting. Ideally, completely independent data sets would be used. Although, the IGAP consortia meta-analysis has sparked exponential increase in GRS studies with an unparalleled resource of genetic information, it has also actualized a need for validation of GRSs in independent data sets.

### Mild cognitive impairment to AD conversion

The GRS results were mixed in predicting AD conversion in participants with MCI^24,25,31^. The most recent study, Tan et al., reported a significant association when comparing the 84^th^ to 16^th^ percentile in a larger sample of more than 1650 individuals. Both Lacour et al. and Rodriguez-Rodriguez et al. reported non-significant associations; however, *APOE* ε4 status did predict AD conversion. Yet, case numbers and power were rather limited in both studies (790 and 118 cases, respectively). More studies are necessary to draw meaningful conclusions regarding the ability of the GRS to predict MCI to AD conversion.

Nonetheless, these results may suggest that other AD susceptibility loci (besides *APOE*) may not be predictors of AD conversion or have miniscule effects. It is also possible that some bias may exist due to the MCI participants that do not develop AD or develop another form of dementia^24^. Another viable explanation is the role of cognitive reserve and environmental factors in AD conversion^35^. Finally, the lack of association may have resulted from chance given the breadth of the confidence intervals.

### GRS compared to APOE ε4

The predictive ability of *APOE* ε4 status to determine AD genetic risk has been well established with one copy and two copies of the *APOE* ε4 allele resulting in a 3-fold and 15-fold increase in risk respectively^36^. Although the GRSs in the included studies are significantly associated with AD diagnosis, it is important to investigate whether a GRS adds to genetic risk stratification above and beyond *APOE* ε4.

The disease prediction accuracy of the GRS (excluding *APOE*) was worse than *APOE* ε4 status. However, when the GRS included *APOE* it did increase the diagnostic accuracy compared to *APOE* ε4 status alone. The best discrimination ability was seen in GRSs that used a large number of SNPs including those in and around the *APOE* locus^10^. It has been estimated that *APOE* ε4 accounts for only 7% of the 65% total potentially non-modifiable risk factors of AD, suggesting further genetic associations beyond *APOE*^37^.

### Implications

GRSs for AD are not currently relevant in a clinical setting, but they have the potential for use as a genetic risk stratification tool in clinical trials as well as future therapeutic interventions. Genetic risk stratification has been used in recent years to individualize therapeutic approaches in several diseases including cancer^38^. In preventable diseases GRSs can help identify those at risk and target preventive strategies accordingly^39^. In the future, genetic risk assessment through a GRS for AD could be integral in personalized medicine regarding AD.

Recently, the National Institute on Aging and Alzheimer’s Association Research Framework has recommended a shift toward a biological definition and the use of biomarkers for in vivo Alzheimer’s diagnosis^40^. GRSs have also shown significant associations to Alzheimer biomarkers including beta amyloid, phosphorylated and total tau^15,21,41,42^, hippocampal and amygdala volume^22,23,33,43,44^, among others. The results are however mixed with some studies reporting non-significant associations between GRSs and beta amyloid and tau^45,46^. The relationship between genetic risk and biomarkers of AD can provide deep insights into disease pathology and overall risk. As the definition of Alzheimer’s shifts to a biological basis, the investigation of genetic risk prediction of AD biomarkers may become even more pertinent.

### Strengths and limitations

There are several limitations to this review. First, the methods, including choice of SNPs, validation samples, and type of reported measure of association varied across the included studies making it difficult to directly compare results. Furthermore, we focused on GRSs based on and validated within datasets including individuals of European descent, limiting the generalizability of the GRSs described. The populations used in the included studies were also often recruited from clinical settings, which therefore might also limit generalizability. As previously mentioned the largest weakness is the overlap between the training and validation sets, that both used IGAP data (Supplementary Table 5).

The included studies did also exhibit many strengths. All studies used thorough genotyping techniques, clinical diagnoses of AD, and proper control selection (if applicable). Statistical methods and study designs were appropriate and several of the more recent studies utilized a longitudinal cohort design providing deeper insight into the relationship between GRS and AD diagnosis.

The information presented in this systematic review is to our knowledge the first analysis of the existing GRSs for AD, further contributing to the AD literature related to genetic risk. The PRISMA guidelines were followed to ensure a rigorous review, selection, and presentation of the included literature. Furthermore, the topic is very timely with most of the results published recently in a field where the identification of genetic risk will continue to be a critical task.

## Conclusion

GRSs including AD associated SNPs seem to be a promising strategy to classify AD genetic risk above and beyond *APOE* ε4, but the ability to predict MCI to AD conversion remains unclear. However, further validation of the GRSs including liberal approaches (not restricted to SNPs reaching genome-wide significance) and population-based prospective studies are warranted to confirm the results obtained with IGAP data. Finally, risk stratification for AD may be further improved by combining *APOE* and GRS status with additional data, such as “environmental” risk factors (including lifestyle factors) or other biomarker data known to be associated with AD risk.

## Electronic supplementary material

Supplementary Information: Alzheimer’s genetic risk score systematic review
