# Supplementary material for: The genetic risk of Alzheimer’s disease beyond APOE ε4: systematic review of Alzheimer’s genetic risk scores
Source: Transl Psychiatry. 2018 Aug 24;8:166. doi: 10.1038/s41398-018-0221-8 (PMC6109140; doi:10.1038/s41398-018-0221-8)
Supplement: Supplementary file 1 — Supplementary Information: Alzheimer’s genetic risk score systematic review [file 41398_2018_221_MOESM1_ESM.docx]

| **Supplementary Table 1: Search strategy results** | | | |
| --- | --- | --- | --- |
| Database | Search strategy | Results | Date |
| PubMed | ((Alzheimer*) AND (("genetic risk prediction" OR "genetic risk prediction model" OR "genetic risk prediction models" OR "genetic risk prediction score" OR "genetic risk prediction studies" OR "genetic risk prediction tool" OR "genetic risk predictions" OR "genetic risk predictor" OR "genetic risk predictors" OR "genetic risk scores" or "genetic risk score" or "genetic risk score model" or "genetic risk status"))) OR ("polygenic risk" OR "polygenic risk score") | 486  160 | 20.09.17  06.04.18 |
| Web of science | TOPIC: (Alzheimer*) AND TOPIC: (genetic risk prediction OR genetic risk prediction model OR genetic risk prediction models OR genetic risk prediction score OR genetic risk prediction studies OR genetic risk prediction tool OR genetic risk predictions OR genetic risk predictor OR genetic risk predictors OR genetic risk scores OR genetic risk score OR genetic risk score model OR genetic risk status OR polygenic risk OR polygenic risk score OR polygenic hazard score) | 1,183  189 | 20.09.17  06.04.18 |

| **Supplementary Table 2: PRISMA 2009 Checklist** | | | | |
| --- | --- | --- | --- | --- |
| **Section/topic** | **#** | **Checklist item** | **Reported on page #** | |
| **TITLE** | | |  | |
| Title | 1 | Identify the report as a systematic review, meta-analysis, or both. | 1 | |
| **ABSTRACT** | | |  | |
| Structured summary | 2 | Provide a structured summary including, as applicable: background; objectives; data sources; study eligibility criteria, participants, and interventions; study appraisal and synthesis methods; results; limitations; conclusions and implications of key findings; systematic review registration number. | 2 | |
| **INTRODUCTION** | | |  | |
| Rationale | 3 | Describe the rationale for the review in the context of what is already known. | 3-4 | |
| Objectives | 4 | Provide an explicit statement of questions being addressed with reference to participants, interventions, comparisons, outcomes, and study design (PICOS). | 4 | |
| **METHODS** | | |  | |
| Protocol and registration | 5 | Indicate if a review protocol exists, if and where it can be accessed (e.g., Web address), and, if available, provide registration information including registration number. | 4 | |
| Eligibility criteria | 6 | Specify study characteristics (e.g., PICOS, length of follow-up) and report characteristics (e.g., years considered, language, publication status) used as criteria for eligibility, giving rationale. | 4 | |
| Information sources | 7 | Describe all information sources (e.g., databases with dates of coverage, contact with study authors to identify additional studies) in the search and date last searched. | 4 | |
| Search | 8 | Present full electronic search strategy for at least one database, including any limits used, such that it could be repeated. | SI-1 | |
| Study selection | 9 | State the process for selecting studies (i.e., screening, eligibility, included in systematic review, and, if applicable, included in the meta-analysis). | 4 | |
| Data collection process | 10 | Describe method of data extraction from reports (e.g., piloted forms, independently, in duplicate) and any processes for obtaining and confirming data from investigators. | 5 | |
| Data items | 11 | List and define all variables for which data were sought (e.g., PICOS, funding sources) and any assumptions and simplifications made. | 5 | |
| Risk of bias in individual studies | 12 | Describe methods used for assessing risk of bias of individual studies (including specification of whether this was done at the study or outcome level), and how this information is to be used in any data synthesis. | 5 | |
| Summary measures | 13 | State the principal summary measures (e.g., risk ratio, difference in means). | 5 | |
| Synthesis of results | 14 | Describe the methods of handling data and combining results of studies, if done, including measures of consistency (e.g., I^2^) for each meta-analysis. | - | |
| **Section/topic** | **#** | **Checklist item** | | **Reported on page #** |
| Risk of bias across studies | 15 | Specify any assessment of risk of bias that may affect the cumulative evidence (e.g., publication bias, selective reporting within studies). | | - |
| Additional analyses | 16 | Describe methods of additional analyses (e.g., sensitivity or subgroup analyses, meta-regression), if done, indicating which were pre-specified. | | - |
| **RESULTS** | | | |  |
| Study selection | 17 | Give numbers of studies screened, assessed for eligibility, and included in the review, with reasons for exclusions at each stage, ideally with a flow diagram. | | 5-6 |
| Study characteristics | 18 | For each study, present characteristics for which data were extracted (e.g., study size, PICOS, follow-up period) and provide the citations. | | 6-7 |
| Risk of bias within studies | 19 | Present data on risk of bias of each study and, if available, any outcome level assessment (see item 12). | | 8-9 |
| Results of individual studies | 20 | For all outcomes considered (benefits or harms), present, for each study: (a) simple summary data for each intervention group (b) effect estimates and confidence intervals, ideally with a forest plot. | | 7-9 |
| Synthesis of results | 21 | Present results of each meta-analysis done, including confidence intervals and measures of consistency. | | - |
| Risk of bias across studies | 22 | Present results of any assessment of risk of bias across studies (see Item 15). | | - |
| Additional analysis | 23 | Give results of additional analyses, if done (e.g., sensitivity or subgroup analyses, meta-regression [see Item 16]). | | 7-9 |
| **DISCUSSION** | | | |  |
| Summary of evidence | 24 | Summarize the main findings including the strength of evidence for each main outcome; consider their relevance to key groups (e.g., healthcare providers, users, and policy makers). | | 9-12 |
| Limitations | 25 | Discuss limitations at study and outcome level (e.g., risk of bias), and at review-level (e.g., incomplete retrieval of identified research, reporting bias). | | 12-14 |
| Conclusions | 26 | Provide a general interpretation of the results in the context of other evidence, and implications for future research. | | 9-14 |
| **FUNDING** | | | |  |
| Funding | 27 | Describe sources of funding for the systematic review and other support (e.g., supply of data); role of funders for the systematic review. | | 14 |

*From:*  Moher D, Liberati A, Tetzlaff J, Altman DG, The PRISMA Group (2009). Preferred Reporting Items for Systematic Reviews and Meta-Analyses: The PRISMA Statement. PLoS Med 6(7): e1000097. doi:10.1371/journal.pmed1000097

| **Supplementary Table 3: SNPs included in GRSs** | | | | | | | | | | | | | | | | | | | | | | |
| --- | --- | --- | --- | --- | --- | --- | --- | --- | --- | --- | --- | --- | --- | --- | --- | --- | --- | --- | --- | --- | --- | --- |
|  | **SNP** | **CHR** | **BP** | **GENE** | ^1^ | ^2^ | ^3^ | ^4^ | ^5^ | ^6^ | ^7^ | ^8^ | ^9^ | ^10^ | ^11^ | ^12^ | ^13^ | ^14^ | ^15^ | ^16^ | ^17^ | ^18^ |
| 1 | **rs11218343** | **11** | 121435587 | SORL1 | x | x | x |  | x | x | x | x | x | x | x |  | x | x |  |  | x |  |
| 2 | **rs1476679** | **7** | 100004446 | ZCWPW1 | x | x | x |  | x | x | x | x | x | x | x |  | x | x |  |  | x |  |
| 3 | **rs7274581** | **20** | 55018260 | CASS4 | x | x | x |  | x | x | x | x | x | x | x |  | x | x |  |  | x |  |
| 4 | **rs6733839** | **2** | 127892810 | BIN1 | x | x | x |  | x | x | x | x | x | x | x |  |  | x |  |  |  |  |
| 5 | **rs28834970** | **8** | 27195121 | PTK2B | x | x | x |  |  | x | x |  | x | x | x |  | x | x |  |  | x |  |
| 6 | **rs2718058** | **7** | 37841534 | NME8 | x | x | x |  |  | x | x |  | x | x | x |  | x | x |  |  | x |  |
| 7 | **rs10498633** | **14** | 92926952 | RINSLC24A4 | x | x |  |  |  | x | x |  | x | x | x |  | x | x |  |  | x |  |
| 8 | **rs17125944** | **14** | 53400629 | FERMT2 | x | x | x |  |  | x | x |  | x | x | x |  | x | x |  |  | x |  |
| 9 | **rs35349669** | **2** | 234068476 | INPP5D | x | x |  |  |  | x | x |  | x | x | x |  | x | x |  |  | x |  |
| 10 | **rs190982** | **5** | 88223420 | MEF2C |  | x |  |  |  | x | x |  | x | x | x |  | x | x |  |  | x |  |
| 11 | **rs10838725** | **11** | 47557871 | CELF1 | x | x | x |  |  | x | x |  | x | x | x |  | x | x |  |  |  |  |
| 12 | **rs10792832** | 11 | 85867875 | PICALM | x | x | x |  |  | x | x |  | x | x | x |  |  | x |  |  |  |  |
| 13 | **rs9331896** | **8** | 27467686 | CLU | x | x | x |  |  | x | x |  | x | x | x |  |  | x |  |  |  |  |
| 14 | **rs6656401** | **1** | 207692049 | CR1 | x | x | x |  |  | x | x |  | x | x | x |  |  | x |  |  | x |  |
| 15 | **rs983392** | **11** | 59923508 | MS4A6A | x | x | x |  |  | x | x |  | x | x | x |  |  | X |  |  |  |  |
| 16 | **rs4147929** | **19** | 1063443 | ABCA7 | x | x | x |  |  | x | x |  | x | x | x |  |  | x |  |  |  |  |
| 17 | **rs11771145** | **7** | 143110762 | EPHA1 | x | x | x |  |  | x | x |  | x | x | x |  |  | x |  |  |  |  |
| 18 | **rs9271192** | **6** | 32578530 | HLADRB5/1 | x |  |  |  |  | x | x |  | x |  | x |  | x | x |  |  |  |  |
| 19 | **rs10948363** | **6** | 47487762 | CD2AP | x | x | x |  |  | x | x |  | x | x | x |  |  | x |  |  | x |  |
| 20 | **rs3865444** | **19** | 51727962 | CD33 |  |  | x |  |  | x | x |  | x |  |  |  | x | x |  |  | x |  |
| 21 | **rs8093731** | **18** | 29088958 | DSG2 |  |  |  |  |  | x | x |  | x |  |  |  |  | x |  |  |  |  |
| 22 | **rs429358/**  **rs7412** | **19** | 45411941/  45412079 | APOE | x |  |  |  | x |  |  | x |  |  |  | x | x |  | x |  |  |  |
| 23 | **rs3851179** | **11** | 86157598 | PICALM |  |  |  |  |  |  |  |  |  |  |  | x | x |  | x |  | x | x |
| 24 | **rs9349407** | **6** | 47485642 | CD2AP |  |  |  |  |  |  |  |  |  |  |  | x | x |  |  |  |  |  |
| 25 | **rs744373** | **2** | 127137039 | BIN1 |  |  |  |  |  |  |  |  |  |  |  |  | x |  |  |  | x | x |
| 26 | **rs7920721** | **10** | 11720308 | CR595071 |  | x |  |  | x |  |  | x |  |  |  |  |  |  |  |  |  |  |
| 27 | **rs3818361** | **1** | 207611623 | CR1 |  |  |  |  |  |  |  |  |  |  |  |  | x |  |  |  |  |  |
| 28 | **rs11136000** | **8** | 2767002 | CLU |  |  |  |  |  |  |  |  |  |  |  |  | x |  | x | x | x | x |
| 29 | **rs3764650** | **19** | 1046521 | ABCA7 |  |  |  |  |  |  |  |  |  |  |  |  | x |  |  |  | x | x |
| 30 | **rs610932** | **11** | 60171834 | MS4A6A |  |  |  |  |  |  |  |  |  |  |  |  | x |  | x |  |  | x |
| 31 | **rs75932628** | **6** | 41161514 | TREM2 | x | x |  |  |  |  |  |  |  |  |  |  | x |  |  |  |  |  |
| 32 | **rs6701713** | **1** | 207612944 | CR1 |  |  |  |  |  |  |  |  |  |  |  | x |  |  |  |  |  |  |
| 33 | **rs9331888** | **8** | 27468862 | CLU |  |  |  |  | x |  |  | x |  |  |  |  |  |  |  |  |  |  |
| 34 | **rs11767557** | **7** | 143412046 | EPHA1 |  |  |  |  |  |  |  |  |  |  |  |  | x |  |  |  |  |  |
| 35 | **rs74615166** | **15** | 64725490 | TRIP4 |  |  |  |  | x |  |  | x |  |  |  |  |  |  |  |  |  |  |
| 36 | **rs3752246** | **19** | 1056492 | ABCA7 |  |  |  |  | x |  |  | x |  |  |  |  |  |  |  |  |  |  |
| 37 | **rs4266886** | **1** | 207685786 | CR1 |  |  |  |  | x |  |  | x |  |  |  |  |  |  |  |  |  |  |
| 38 | **rs61822977** | **1** | 207796065 | CR1 |  |  |  |  | x |  |  | x |  |  |  |  |  |  |  |  |  |  |
| 39 | **rs1020278** | **2** | 234003117 | INPP5D |  |  |  |  | x |  |  | x |  |  |  |  |  |  |  |  |  |  |
| 40 | **rs1768208** | **3** | 39481512 | MOBP |  |  |  |  |  |  |  |  |  |  |  | x |  |  |  |  |  |  |
| 41 | **rs6599389** | **4** | 945325 | TMEM175 |  |  |  |  |  |  |  |  |  |  |  | x |  |  |  |  |  |  |
| 42 | **rs115124923** | **6** | 32510482 | HLA-DRB5 |  |  |  |  | x |  |  | x |  |  |  |  |  |  |  |  |  |  |
| 43 | **rs115675626** | **6** | 32669833 | HLA-DQB1 |  |  |  |  | x |  |  | x |  |  |  |  |  |  |  |  |  |  |
| 44 | **rs4504469** | **6** | 24588656 | KIAA0319 |  |  |  |  |  |  |  |  |  |  |  | x |  |  |  |  |  |  |
| 45 | **rs1109581** | **6** | 47678182 | GPR115 |  |  |  |  | x |  |  | x |  |  |  |  |  |  |  |  |  |  |
| 46 | **rs1799945** | **6** | 26090951 | HFE |  |  |  |  |  |  |  |  |  |  |  | x |  |  |  |  |  |  |
| 47 | **rs17236239** | **7** | 147885213 | CNTNAP2 |  |  |  |  |  |  |  |  |  |  |  | x |  |  |  |  |  |  |
| 48 | **rs17265593** | **7** | 37619922 | BC0433356 |  |  |  |  | x |  |  | x |  |  |  |  |  |  |  |  |  |  |
| 49 | **rs2597283** | **7** | 37690507 | BC0433356 |  |  |  |  | x |  |  | x |  |  |  |  |  |  |  |  |  |  |
| 50 | **rs78571833** | **7** | 143122924 | AL833583 |  |  |  |  | x |  |  | x |  |  |  |  |  |  |  |  |  |  |
| 51 | **rs12679874** | **8** | 27230819 | PTK2B |  |  |  |  | x |  |  | x |  |  |  |  |  |  |  |  |  |  |
| 52 | **rs2741342** | **8** | 27330096 | CHRNA2 |  |  |  |  | x |  |  | x |  |  |  |  |  |  |  |  |  |  |
| 53 | **rs7831810** | **8** | 27430506 | CLU |  |  |  |  | x |  |  | x |  |  |  |  |  |  |  |  |  |  |
| 54 | **rs1532277** | **8** | 27466181 | CLU |  |  |  |  | x |  |  | x |  |  |  |  |  |  |  |  |  |  |
| 55 | **rs3849942** | **9** | 27543283 | C9ORF72 |  |  |  |  |  |  |  |  |  |  |  | x |  |  |  |  |  |  |
| 56 | **rs3740688** | **11** | 47380340 | SPI1 |  |  |  |  | x |  |  | x |  |  |  |  |  |  |  |  |  |  |
| 57 | **rs7116190** | **11** | 59964992 | MS4A6A |  |  |  |  | x |  |  | x |  |  |  |  |  |  |  |  |  |  |
| 58 | **rs526904** | **11** | 85811354 | PICALM |  |  |  |  | x |  |  | x |  |  |  |  |  |  |  |  |  |  |
| 59 | **rs543293** | **11** | 85820077 | PICALM |  |  |  |  | x |  |  | x |  |  |  |  |  |  |  |  |  |  |
| 60 | **rs1799913** | **11** | 18025708 | TPH1 |  |  |  |  |  |  |  |  |  |  |  | x |  |  |  |  |  |  |
| 61 | **rs12285364** | **11** | 121522517 | SORL1 |  |  |  |  |  |  |  |  |  |  |  | x |  |  |  |  |  |  |
| 62 | **rs2070045** | **11** | 121577381 | SORL1 |  |  |  |  |  |  |  |  |  |  |  | x |  |  |  |  |  |  |
| 63 | **rs6572869** | **14** | 53353454 | FERMT2 |  |  |  |  | x |  |  | x |  |  |  |  |  |  |  |  |  |  |
| 64 | **rs12590273** | **14** | 92934120 | SLC24A4 |  |  |  |  | x |  |  | x |  |  |  |  |  |  |  |  |  |  |
| 65 | **rs7145100** | **14** | 107160690 | abParts |  |  |  |  | x |  |  | x |  |  |  |  |  |  |  |  |  |  |
| 66 | **rs2020942** | **17** | 30219896 | SLC6A4 |  |  |  |  |  |  |  |  |  |  |  | x |  |  |  |  |  |  |
| 67 | **rs2526378** | **17** | 56404349 | BVRAP1 |  |  |  |  | x |  |  | x |  |  |  |  |  |  |  |  |  |  |
| 68 | **rs4130047** | **18** | 43098270 | RIT2 |  |  |  |  |  |  |  |  |  |  |  | x |  |  |  |  |  |  |
| 69 | **rs117481827** | **19** | 1021627 | C19orf6 |  |  |  |  | x |  |  | x |  |  |  |  |  |  |  |  |  |  |
| 70 | **rs7408475** | **19** | 1050130 | ABCA7 |  |  |  |  | x |  |  | x |  |  |  |  |  |  |  |  |  |  |
| 71 | **rs4680** | **22** | 19963748 | COMT |  |  |  |  |  |  |  |  |  |  |  | x |  |  |  |  |  |  |
| 72 | **rs1320490** | **-** | - | CDC42BPA |  |  |  |  |  |  |  |  |  |  |  | x |  |  |  |  |  |  |
| 73 | **rs8053211** | - | - | ATP2C2 |  |  |  |  |  |  |  |  |  |  |  | x |  |  |  |  |  |  |
| 74 | **rs7814569** | **-** | - | TPD52 |  |  |  |  |  |  |  |  |  |  |  | x |  |  |  |  |  |  |
| 75 | **rs11186856** | **-** | - | CPE |  |  |  |  |  |  |  |  |  |  |  | x |  |  |  |  |  |  |
| 76 | **rs1539053** | **1** | 57634035 | DAB1 |  |  |  |  |  |  |  |  |  |  |  |  |  |  | x | x |  |  |
| 77 | **rs1408077** | **-** | - | - |  |  |  |  |  |  |  |  |  |  |  |  |  |  | x |  |  | x |
| 78 | **rs7561528** | **2** | 127132061 | LOC105373605 |  |  |  |  |  |  |  |  |  |  |  |  |  |  | x | x |  |  |
| 79 | **rs11894266** | **-** | - | - |  |  |  |  |  |  |  |  |  |  |  |  |  |  | x | x |  |  |
| 80 | **rs9446432** | **-** | - | - |  |  |  |  |  |  |  |  |  |  |  |  |  |  | x | x |  |  |
| 81 | **rs9384428** | **-** | - | - |  |  |  |  |  |  |  |  |  |  |  |  |  |  | x | x |  |  |
| 82 | **rs2582367** | **-** | - | - |  |  |  |  |  |  |  |  |  |  |  |  |  |  | x |  |  |  |
| 83 | **rs1157242** | **-** | - | - |  |  |  |  |  |  |  |  |  |  |  |  |  |  | x | x |  |  |
| 84 | **rs11827375** | **-** | - | - |  |  |  |  |  |  |  |  |  |  |  |  |  |  | x |  |  |  |
| 85 | **rs10501927** | **-** | - | - |  |  |  |  |  |  |  |  |  |  |  |  |  |  | x | x |  |  |
| 86 | **rs2965101** | **-** | - | - |  |  |  |  |  |  |  |  |  |  |  |  |  |  | x |  |  |  |
| 87 | **rs2927438** | **19** | 44738850 | LOC107985305 - BCL3 |  |  |  |  |  |  |  |  |  |  |  |  |  |  | x |  |  |  |
| 88 | **rs10402271** | **-** | - | - |  |  |  |  |  |  |  |  |  |  |  |  |  |  | x |  |  |  |
| 89 | **rs1871047** | **-** | - | - |  |  |  |  |  |  |  |  |  |  |  |  |  |  | x |  |  |  |
| 90 | **rs377702** | **-** | - | - |  |  |  |  |  |  |  |  |  |  |  |  |  |  | x |  |  |  |
| 91 | **rs12610605** | **-** | - | - |  |  |  |  |  |  |  |  |  |  |  |  |  |  | x |  |  |  |
| 92 | **rs6859** | **19** | 44878777 | PVRL2 |  |  |  |  |  |  |  |  |  |  |  |  |  |  | x |  |  |  |
| 93 | **rs157580** | **19** | 44892009 | APOE/TOMM40 |  |  |  |  |  |  |  |  |  |  |  |  |  |  | x |  |  |  |
| 94 | **rs2075650** | **19** | 44892362 | APOE/TOMM40 |  |  |  |  |  |  |  |  |  |  |  |  |  |  | x |  |  |  |
| 95 | **rs8106922** | **-** | - | - |  |  |  |  |  |  |  |  |  |  |  |  |  |  | x |  |  |  |
| 96 | **rs439401** | **19** | 44911194 | APOE - APOC1P1 |  |  |  |  |  |  |  |  |  |  |  |  |  |  | x |  |  |  |
| 97 | **rs5167** | **19** | 44945208 | APOC4 – APOC2 |  |  |  |  |  |  |  |  |  |  |  |  |  |  | x |  |  |  |
| 98 | **rs1048699** | **-** | - | - |  |  |  |  |  |  |  |  |  |  |  |  |  |  | x |  |  |  |
| 99 | **rs676309** | **-** | - | - |  |  |  |  |  |  |  |  |  |  |  |  |  |  |  | x |  |  |
| 100 | **rs662196** | **-** | - | - |  |  |  |  |  |  |  |  |  |  |  |  |  |  |  | x |  |  |
| 101 | **rs11952762** | **-** | - | - |  |  |  |  |  |  |  |  |  |  |  |  |  |  |  | x |  |  |
| 102 | **rs12201301** | **-** | - | - |  |  |  |  |  |  |  |  |  |  |  |  |  |  |  | x |  |  |
| 103 | **rs10499889** | **-** | - | - |  |  |  |  |  |  |  |  |  |  |  |  |  |  |  | x |  |  |
| 104 | **rs8055533** | **-** | - | - |  |  |  |  |  |  |  |  |  |  |  |  |  |  |  | x |  |  |
| 105 | **rs10808026** | **-** | - | - |  |  |  |  |  |  |  |  |  |  |  |  |  |  |  |  | x |  |
| 106 | **rs4938933** | **11** | 60266956 | MS4A4A |  |  |  |  |  |  |  |  |  |  |  |  |  |  |  |  | x |  |
| 107 | **rs9296559** | **6** | 47484534 | CD2AP |  |  |  |  |  |  |  |  |  |  |  |  |  |  |  |  |  | x |
| 108 | **rs670139** | **-** | - | - |  |  |  |  |  |  |  |  |  |  |  |  |  |  |  |  |  | x |
| 109 | **rs111418223** | **6** |  | HLA-DRB1/5 |  | x | x |  |  |  |  |  |  |  |  |  |  |  |  |  |  |  |
| 110 | **rs13113697** | **4** |  | HS3ST1 |  | x |  |  |  |  |  |  |  |  |  |  |  |  |  |  |  |  |
| 111 | **rs118172952** | **17** |  | KANSLI |  | x |  |  |  |  |  |  |  |  |  |  |  |  |  |  |  |  |
| 112 | **rs190982** | **5** | 88223420 | INPP5D | x |  |  |  |  |  |  |  |  |  |  |  |  |  |  |  |  |  |

Note: For both Escott-Price et al. studies and Lupton et al., only SNPs included in the conservative GRSs are presented ^7, 11, 14^ SNP information for Chaudhury et al. was not available^4^.

BP: base position

GRSs: Genetic risk scores

SNP: single nucleotide polymorphism

| **Supplementary Table 4: Quality rating of included studies – modified Newcastle-Ottawa scale** | | | | | | | | | | |  |
| --- | --- | --- | --- | --- | --- | --- | --- | --- | --- | --- | --- |
|  | **Selection** | | | | **Comparability** | | **Exposure (Genetic Risk)** | | | | **Total**  **(of 9)** |
| *Case-control studies* | | | | | | | | | | |  |
| Study | Case Definition | Representative | Control selection | Control Definition | Controls for age, sex | Controls for *APOE* (or includes) | Genetic risk | Same method case/cont. | | Missing data rate |  |
| Cruchaga,, 2018 | ✓ | ✓ | ✓ | ✓ | ✓ | ✓ | ✓ | ✓ | |  | 8 |
| Chaudhury, 2017 | ✓ |  | ✓ | ✓ |  |  | ✓ | ✓ | |  | 5 |
| Tosto, 2017 ^6^ | ✓ |  | ✓ | ✓ | ✓ | ✓ | ✓ | ✓ | |  | 7 |
| Escott-Price, 2017 ^7^ | ✓ | ✓ | ✓ | ✓ |  | ✓ | ✓ | ✓ | |  | 7 |
| Tosto, 2016 ^9^ | ✓ |  |  | ✓ | ✓ | ✓ | ✓ | ✓ | |  | 6 |
| Lupton, 2016 ^11^ | ✓ | ✓ | ✓ | ✓ | ✓ |  | ✓ | ✓ | |  | 7 |
| Yokoyama, 2015 ^12^ | ✓ |  | ✓ | ✓ |  | ✓ | ✓ | ✓ | |  | 6 |
| Sleegers, 2015 ^13^ | ✓ | ✓ | ✓ | ✓ | ✓ | ✓ | ✓ | ✓ | |  | 8 |
| Escott-Price, 2015 ^14^ | ✓ | ✓ | ✓ | ✓ | ✓ | ✓ | ✓ | ✓ | |  | 8 |
| Biffi, 2010 ^16^ | ✓ | ✓ | ✓ | ✓ | ✓ | ✓ | ✓ | ✓ | |  | 8 |
| *Cohort studies* | | | | | | | | | | |  |
| Study | Representative | Sample size | Genetic risk | No outcome | Controls for age, sex | Controls for APOE (or includes) | Outcome assessment | FU length | Ad. FU cohorts | |  |
| Ahmad, 2018 | ✓ | ✓ | ✓ | ✓ | ✓ | ✓ | ✓ | ✓ |  | | 8 |
| Van der Lee, 2018 | ✓ | ✓ | ✓ | ✓ | ✓ | ✓ | ✓ | ✓ |  | | 8 |
| Tan, 2017 | ✓ | ✓ | ✓ | ✓ | ✓ | ✓ | ✓ |  |  | | 7 |
| Lacour, 2017 ^17^ | ✓ | ✓ | ✓ | ✓ | ✓ | ✓ | ✓ | ✓ |  | | 8 |
| Desikan, 2017 ^8^ | ✓ | ✓ | ✓ | ✓ | ✓ | ✓ | ✓ | ✓ |  | | 8 |
| Chouraki, 2016 ^10^ | ✓ | ✓ | ✓ | ✓ | ✓ | ✓ | ✓ | ✓ |  | | 8 |
| Rodriguez-Rodriguez, 2013 ^18^ | ✓ |  | ✓ | ✓ | ✓ | ✓ | ✓ | ✓ |  | | 7 |
| *Cross sectional* | | | | | | | | | | |  |
| Study | Representative | Sample size | Genetic risk | Missing data | Controls for age, sex | Controls for *APOE* (or includes) | Outcome assessment | | Statistical method | |  |
| Sabuncu, 2012 ^15^ | ✓ |  | ✓ |  | ✓ | ✓ | ✓ | ✓ |  | | 6 |

Ad., adequacy

*APOE*: apolipoprotein E

FU, follow-up

| **Supplementary Table 5:** **Studies with IGAP overlap in training and validation sets** | | | | |
| --- | --- | --- | --- | --- |
| First Author, Year | IGAP Overlap  (Yes/No) | Overlap (%) | Discussed  (Yes/No) | Handled |
| Ahmad, 2018^1^ | ✓ | 625 Cases (49%) | ✓ | Additional analysis excluding overlap |
| Van der Lee, 2018^2^ | ✓ | - | - | - |
| Cruchaga, 2018^3^ | ✓ | - | ✓ | Additional analysis excluding overlap |
| Chaudhury, 2018^4^ | X | - | - | - |
| Tan, 2017^5^ | ✓ | - | - | - |
| Tosto, 2017^6^ | ✓ | - | ✓ | - |
| Escott-Price, 2017^7^ | ✓ | 3% | ✓ | Statistically accounted for overfitting |
| Desikan, 2017^8^ | ✓ | - | - | - |
| Tosto, 2016^9^ | ✓ | - | - | - |
| Chouraki, 2016^10^ | ✓ | - | ✓ | Additional analysis excluding overlap |
| Lupton, 2016^11^ | X | - | - |  |
| Sleegers, 2015^13^ | ✓ | - | - | - |
| Escott-Price, 2015^14^ | ✓ | - | ✓ | Excluded overlapping subset in training set |
| Lacour, 2017^17^ | X | - | - | - |

**References**

1. Ahmad S, Bannister C, van der Lee SJ, Vojinovic D, Adams HHH, Ramirez A *et al.* Disentangling the biological pathways involved in early features of Alzheimer's disease in the Rotterdam Study. *Alzheimer's & dementia : the journal of the Alzheimer's Association* 2018.

2. van der Lee SJ, Wolters FJ, Ikram MK, Hofman A, Ikram MA, Amin N *et al.* The effect of APOE and other common genetic variants on the onset of Alzheimer's disease and dementia: a community-based cohort study. *The Lancet Neurology* 2018.

3. Cruchaga C, Del-Aguila JL, Saef B, Black K, Fernandez MV, Budde J *et al.* Polygenic risk score of sporadic late-onset Alzheimer's disease reveals a shared architecture with the familial and early-onset forms. *Alzheimer's & dementia : the journal of the Alzheimer's Association* 2018; **14**(2)**:** 205-214.

4. Chaudhury S, Patel T, Barber IS, Guetta-Baranes T, Brookes KJ, Chappell S *et al.* Polygenic risk score in postmortem diagnosed sporadic early-onset Alzheimer's disease. *Neurobiology of aging* 2018; **62:** 244.e241-244.e248.

5. Tan CH, Fan CC, Mormino EC, Sugrue LP, Broce IJ, Hess CP *et al.* Polygenic hazard score: an enrichment marker for Alzheimer's associated amyloid and tau deposition. *Acta neuropathologica* 2018; **135**(1)**:** 85-93.

6. Tosto G, Bird TD, Tsuang D, Bennett DA, Boeve BF, Cruchaga C *et al.* Polygenic risk scores in familial Alzheimer disease. *Neurology* 2017; **88**(12)**:** 1180-1186.

7. Escott-Price V, Myers AJ, Huentelman M, Hardy J. Polygenic Risk Score Analysis of Pathologically Confirmed Alzheimer's Disease. *Annals of neurology* 2017.

8. Desikan RS, Fan CC, Wang Y, Schork AJ, Cabral HJ, Cupples LA *et al.* Genetic assessment of age-associated Alzheimer disease risk: Development and validation of a polygenic hazard score. *PLoS Med* 2017; **14**(3)**:** e1002258.

9. Tosto G, Bird TD, Bennett DA, Boeve BF, Brickman AM, Cruchaga C *et al.* The Role of Cardiovascular Risk Factors and Stroke in Familial Alzheimer Disease. *JAMA neurology* 2016; **73**(10)**:** 1231-1237.

10. Chouraki V, Reitz C, Maury F, Bis JC, Bellenguez C, Yu L *et al.* Evaluation of a Genetic Risk Score to Improve Risk Prediction for Alzheimer's Disease. *Journal of Alzheimer's disease : JAD* 2016; **53**(3)**:** 921-932.

11. Lupton MK, Strike L, Hansell NK, Wen W, Mather KA, Armstrong NJ *et al.* The effect of increased genetic risk for Alzheimer's disease on hippocampal and amygdala volume. *Neurobiology of aging* 2016; **40:** 68-77.

12. Yokoyama JS, Bonham LW, Sears RL, Klein E, Karydas A, Kramer JH *et al.* Decision tree analysis of genetic risk for clinically heterogeneous Alzheimer's disease. *BMC neurology* 2015; **15:** 47.

13. Sleegers K, Bettens K, De Roeck A, Van Cauwenberghe C, Cuyvers E, Verheijen J *et al.* A 22-single nucleotide polymorphism Alzheimer's disease risk score correlates with family history, onset age, and cerebrospinal fluid Abeta42. *Alzheimer's & dementia : the journal of the Alzheimer's Association* 2015; **11**(12)**:** 1452-1460.

14. Escott-Price V, Sims R, Bannister C, Harold D, Vronskaya M, Majounie E *et al.* Common polygenic variation enhances risk prediction for Alzheimer's disease. *Brain : a journal of neurology* 2015; **138**(Pt 12)**:** 3673-3684.

15. Sabuncu MR, Buckner RL, Smoller JW, Lee PH, Fischl B, Sperling RA. The association between a polygenic Alzheimer score and cortical thickness in clinically normal subjects. *Cerebral cortex (New York, NY : 1991)* 2012; **22**(11)**:** 2653-2661.

16. Biffi A, Anderson CD, Desikan RS, Sabuncu M, Cortellini L, Schmansky N *et al.* Genetic variation and neuroimaging measures in Alzheimer disease. *Archives of neurology* 2010; **67**(6)**:** 677-685.

17. Lacour A, Espinosa A, Louwersheimer E, Heilmann S, Hernandez I, Wolfsgruber S *et al.* Genome-wide significant risk factors for Alzheimer's disease: role in progression to dementia due to Alzheimer's disease among subjects with mild cognitive impairment. *Molecular psychiatry* 2017; **22**(1)**:** 153-160.

18. Rodriguez-Rodriguez E, Sanchez-Juan P, Vazquez-Higuera JL, Mateo I, Pozueta A, Berciano J *et al.* Genetic risk score predicting accelerated progression from mild cognitive impairment to Alzheimer's disease. *Journal of neural transmission (Vienna, Austria : 1996)* 2013; **120**(5)**:** 807-812.
